# Editorial: Neuromodulation using spatiotemporally complex patterns

**DOI:** 10.3389/fninf.2024.1454834

**Published:** 2024-08-05

**Authors:** Peter A. Tass, Hemant Bokil

**Affiliations:** ^1^Department of Neurosurgery, Stanford University, Stanford, CA, United States,; ^2^Boston Scientific Neuromodulation, Valencia, CA, United States

**Keywords:** neuromodulation, deep brain stimulation, patterned stimulation, coordinated reset stimulation (CRS), plasticity, multi-channel stimulation

Standard high-frequency deep brain stimulation (DBS) is an established therapy for the treatment of Parkinson's disease (PD) (Lozano et al., 2019). However, there is still a significant clinical need for further improvement, as DBS may cause side effects and its therapeutic effects may be limited, in particular, regarding axial symptoms (Baizabal-Carvallo and Jankovic, 2016; Lozano et al., 2019). The articles in this Research Topic highlight that stimulation with spatiotemporal patterns may engage the nervous system in fundamentally different ways than can be achieved with conventional single-frequency stimulation.

Theta burst stimulation (TBS) was initially developed for transcranial magnetic stimulation, especially to induce long-lasting modulation of motor networks (Huang et al., 2005). Later, this stimulus pattern was also applied to DBS. In a randomized, double-blind, clinical short-term trial, Horn et al. (2020) compared two types of TBS unilaterally delivered to the STN with standard unilateral DBS. Their results demonstrated safety and efficacy in this acute (20-30 min) setting, but no long-lasting aftereffects. Sáenz-Farret et al. (2021) studied safety and efficacy of chronically applied bilateral low intra-burst frequency TBS [as introduced by Horn et al. (2020)] in eight PD and one essential tremor patient. In seven patients TBS had to be discontinued due to side effects. Gülke et al. performed an analogous short-term study to test bilateral STN TBS under the same acute conditions and retrospectively combined their data with the data by Horn et al. (2020). Both unilateral and bilateral STN TBS reduced motor scores, where bilateral TBS did not lead to significant additive benefit. Note that the parameters for TBS used in these studies were not all the same which may explain the differences in results. In particular, Sáenz-Farret et al. (2021) utilized a lower intra-burst frequency and twice the inter-burst period as that used in Gülke et al..

Coordinated Reset (CR) stimulation is a patterned multi-site stimulation technique that was computationally developed to specifically counteract abnormal neuronal synchrony by demand-controlled delivery of stimuli that cause robust desynchronization, thereby overcoming limitations of phase-dependent stimulation (Tass, 2003). Using spike-timing dependent plasticity (STDP) (Markram et al., 1997) in a variety of neuronal network models, CR stimulation turned out to induce cumulative and long-lasting desynchronizing effects, by inducing an unlearning of abnormal synaptic connectivity (Tass and Majtanik, 2006; Hauptmann and Tass, 2009). These computationally predicted, cumulative and weeks-long stimulus after-effects, very different compared to what was known from standard DBS, were verified in MPTP Parkinsonian monkeys (Tass et al., 2012; Wang et al., 2016, 2022; Bore et al., 2022) and human PD patients (Adamchic et al., 2014). Bosley et al. study the impact of CR-DBS delivered to the STN specifically on impaired gait in MPTP Parkinsonian monkeys. Their results show that CR-DBS can improve Parkinsonian gait. Kromer et al. present a computational model of the STN-GPe circuit and investigate how connectivity changes affect evoked responses and, hence, can be used to probe functional channels in the basal ganglia by means of a suggested two-site stimulation protocol. These results may lead to calibration techniques for CR-DBS enabled by implantable pulse generators that are able to sense.

In their review article, Najera et al. summarize alternative DBS stimulation approaches and their potential clinical applications. By the same token, in a review article, Cota et al. discuss standard and alternative brain stimulation techniques, including non-periodic stimulation. Different plasticity as well as compensatory mechanisms appear to play crucial roles in Parkinson's disease (Blandini et al., 2000; van Nuenen et al., 2012; Madadi Asl et al., 2022). Accordingly, in an opinion article, Asp et al. stress the importance of neuroplasticity as a key target for the development of novel stimulation techniques.

Adaptive deep brain stimulation (aDBS) has a long history, dating back to the 1980s (Krauss et al., 2021). One goal of aDBS is to reduce side effects by reducing stimulation current. In a computational study, Bahadori-Jahromi et al. compare standard DBS with aDBS with amplitude modulation in a cortico-BG-thalamic network. In their model, aDBS outperformed standard DBS with respect to reduction of beta band oscillations, restoring fidelity of thalamic throughput and overall stimulation current.

As shown computationally, properly timed multi-channel and multi-site stimulation can significantly reshape connectivity, thereby inducing long-lasting activity changes (Khaledi-Nasab et al., 2022; Kromer and Tass, 2022; Madadi Asl et al., 2023). Depending on the condition, restoring function may require to up- or down-regulate specific connections within and/or between brain areas and corresponding patterns of synchrony. In an N-of-1 case report study, Omae et al. use amplitude-modulated transcranial alternating current stimulation (AM-tACS) (Witkowski et al., 2016; Negahbani et al., 2018) to enhance low beta phase synchrony between Broca's area and the right homotopic area with the intend to improve language function in a patient with chronic post-stroke aphasia. Favorable electrophysiological outcomes and clinical benefits indicate that this approach deserves further clinical testing.

In mouse models of Alzheimer's disease, entrainment by gamma (40 Hz) rhythmic light flicker enabled to attenuate pathological processes associated with Alzheimer's disease (Iaccarino et al., 2016; Adaikkan et al., 2019). To computationally study the electrophysiology of gamma flicker entrainment, Wang et al. propose a neural network model for thalamocortical oscillations (TCOs) and computationally studied the impact of light flicker stimulation with different parameters in dependence on different thalamocortical oscillatory states. They revealed state-dependent stimulus responses that may inform future experiments.

EEG plays an important role in monitoring treatment effects and providing feedback for closed-loop stimulation techniques. Motivated by deep learning and stack generalization theory, Zhang et al. propose a novel method for the recognition of epileptic EEG signals: deep extreme learning machine (DELM) which consists of several independent, hierarchically aligned extreme learning machine (ELM) modules. They compared DELM with ELM alone, by applying it to the publicly available EEG data set from the Department of Epileptology at Bonn University, Germany (Andrzejak et al., 2001). In this comparison DELM outperformed ELM regarding accuracy and computing time.

Modern neuromodulation devices, increasingly capable of complex stimulation patterns, and modern tools for data analysis may pave the way for leveraging the potential of novel patterned and multichannel stimulation approaches for clinical use.

## Author contributions

PT: Writing – original draft, Writing – review & editing. HB: Writing – review & editing.

## References

[B1] Adaikkan C. Middleton S. J. Marco A. Pao P. C. Mathys H. Kim D. N.. (2019). Gamma entrainment binds higher-order brain regions and offers neuroprotection. Neuron 102, 929–943.e8. 10.1016/j.neuron.2019.04.011 31076275 PMC6697125

[B2] Adamchic I. Hauptmann C. Barnikol U. B. Pawelcyk N. Popovych O. V. Barnikol T.. (2014). Coordinated reset neuromodulation for Parkinson's disease: proof-of-concept study. Mov. Disord. 29, 1679–1684. 10.1002/mds.25923 24976001 PMC4282372

[B3] Andrzejak R. G. Lehnertz K. Mormann F. Rieke C. David P. Elger C. E.. (2001). Indications of non-linear deterministic and finite-dimensional structures in time series of brain electrical activity: dependence on recording region and brain state. Phys. Rev. E Stat. Nonlin. Soft. Matter. Phys. 64:e061907. 10.1103/PhysRevE.64.061907 11736210

[B4] Baizabal-Carvallo J. F. Jankovic J. (2016). Movement disorders induced by deep brain stimulation. Parkinsonism Relat. Disord. 25, 1–9. 10.1016/j.parkreldis.2016.01.014 26806438

[B5] Blandini F. Nappi G. Tassorelli C. Martignoni E. (2000). Functional changes of the basal ganglia circuitry in Parkinson's disease. Progr. Neurobiol. 62, 63–88. 10.1016/S0301-0082(99)00067-2 10821982

[B6] Bore J. C. Campbell B. A. Cho H. Pucci F. Gopalakrishnan R. Machado A. G.. (2022). Long-lasting effects of subthalamic nucleus coordinated reset deep brain stimulation in the non-human primate model of parkinsonism: a case report. Brain Stimul. 15, 598–P600. 10.1016/j.brs.2022.04.005 35405326 PMC10203883

[B7] Hauptmann C. Tass P. A. (2009). Cumulative and after-effects of short and weak coordinated reset stimulation - a modeling study. J. Neural Eng. 6:016004. 10.1088/1741-2560/6/1/016004 19141875

[B8] Horn M. A. Gulberti A. Gülke E. Buhmann C. Gerloff C. Moll C. K. E.. (2020). A new stimulation mode for deep brain stimulation in Parkinson's disease: theta burst stimulation. Mov. Disord. 35, 1471–1475. 10.1002/mds.28083 32357269

[B9] Huang Y.-Z. Edwards M. J. Rounis E. Bhatia K. P. Rothwell J. C. (2005) Theta burst stimulation of the human motor cortex. Neuron 45, 201–206. 10.1016/j.neuron.2004.12.033 15664172

[B10] Iaccarino H. F. Singer A. C. Martorell A. J. Rudenko A. Gao F. Gillingham T. Z.. (2016). Gamma frequency entrainment attenuates amyloid load and modifies microglia. Nature 540, 230–251. 10.1038/nature20587 27929004 PMC5656389

[B11] Khaledi-Nasab A. Kromer J. A. Tass P. A. (2022) Long-lasting desynchronization of plastic neuronal networks by double-random coordinated reset stimulation. Front. Netw. Physiol. 2:864859. 10.3389/fnetp.2022.864859 36926109 PMC10013062

[B12] Krauss J. K. Lipsman N. Aziz T. Boutet A. Brown P. Woo Chang J.. (2021). Technology of deep brain stimulation: current state and future directions. Nat. Rev. Neurol. 17, 75–87. 10.1038/s41582-020-00426-z 33244188 PMC7116699

[B13] Kromer J. A. Tass P. A. (2022). Synaptic reshaping of plastic neuronal networks by periodic multichannel stimulation with single-pulse and burst stimuli. PLoS Comput. Biol. 18:e1010568. 10.1371/journal.pcbi.1010568 36327232 PMC9632832

[B14] Lozano A. M. Lipsman N. Bergman H. Brown P. Chabardes S. Chang J. W.. (2019). Deep brain stimulation: current challenges and future directions. Nat. Rev. Neurol. 15, 148–160. 10.1038/s41582-018-0128-2 30683913 PMC6397644

[B15] Madadi Asl M. Vahabie A.-H. Valizadeh A. Tass P. A. (2023). Decoupling of interacting neuronal populations by time-shifted stimulation through spike-timing-dependent plasticity. PLoS Comput. Biol. 19:e1010853. 10.1371/journal.pcbi.1010853 36724144 PMC9891531

[B16] Madadi Asl M. Vahabie A. H. Valizadeh A. Tass P. A. (2022). Spike- timing-dependent plasticity mediated by dopamine and its role in Parkinson's disease pathophysiology. Front. Netw. Physiol. 2, 1–18. 10.3389/fnetp.2022.817524 36926058 PMC10013044

[B17] Markram H. Lübke J. Frotscher M. Sakmann B. (1997). Regulation of synaptic efficacy by coincidence of postsynaptic aps and Epsps. Science 275, 213–215. 10.1126/science.275.5297.213 8985014

[B18] Negahbani E. Kasten F. H. Herrmann C. S. Fröhlich F. (2018). Targeting alpha-band oscillations in a cortical model with amplitude-modulated high-frequency transcranial electric stimulation. Neuroimage 173, 3–12. 10.1016/j.neuroimage.2018.02.005 29427848 PMC5911251

[B19] Sáenz-Farret M. Loh A. Boutet A. Germann J. Elias G. J. B. Kalia S. K.. (2021). Theta burst deep brain stimulation in movement disorders. Mov. Disord. Clin. Pract. 8, 282–285. 10.1002/mdc3.13130 33681412 PMC7906022

[B20] Tass P. A. (2003). A model of desynchronizing deep brain stimulation with a demand-controlled coordinated reset of neural subpopulations. Biol. Cybern. 89, 81–88. 10.1007/s00422-003-0425-7 12905037

[B21] Tass P. A. Majtanik M. (2006). Long-term anti-kindling effects of desynchronizing brain stimulation: a theoretical study. Biol. Cybern. 94, 58–66. 10.1007/s00422-005-0028-6 16284784

[B22] Tass P. A. Qin L. Hauptmann C. Dovero S. Bezard E. Boraud T.. (2012). Coordinated reset has sustained after-effects in Parkinsonian monkeys. Ann. Neurol. 72, 816–820. 10.1002/ana.23663 23280797

[B23] van Nuenen B. F. L. Helmich R. C. Buenen N. van de Warrenburg B. P. C. Bloem B. R. Toni I.. (2012). Compensatory activity in the extrastriate body area of Parkinson's disease patients. J. Neurosci. 32, 9546–53. 10.1523/JNEUROSCI.0335-12.2012 22787040 PMC6622256

[B24] Wang J. Fergus S. P. Johnson L. A. Nebeck S. D. Zhang J. Kulkarni S.. (2022). Shuffling improves the acute and carryover effect of subthalamic coordinated reset deep brain stimulation. Front. Neurol. 13:716046. 10.3389/fneur.2022.716046 35250798 PMC8894645

[B25] Wang J. Nebeck S. Muralidharan A. Johnson M. D. Vitek J. L. Baker K. B.. (2016). Coordinated reset deep brain stimulation of subthalamic nucleus produces long-lasting, dose-dependent motor improvements in the 1- methyl-4-phenyl-1,2,3,6-tetrahydropyridine non-human primate model of parkinsonism. Brain Stimul. 9, 609–617. 10.1016/j.brs.2016.03.014 27151601 PMC10226766

[B26] Witkowski M. Garcia-Cossio E. Chander B. S. Braun C. Birbaumer N. Robinson S. E.. (2016). Mapping entrained brain oscillations during transcranial alternating current stimulation (tACS). Neuroimage 140, 89–98. 10.1016/j.neuroimage.2015.10.024 26481671

